# Growth Performance, Dietary Enzyme Profiling, and Antioxidant-Induced Immunity of *Crassostrea belcheri* Fed With Microalgal Diet

**DOI:** 10.1155/anu/5513113

**Published:** 2025-02-06

**Authors:** Mahima Ranjan Acharjee, Md. Saddam Hossain, Subeda Newase, Trina Das, Mohammad Ekramul Haque, Sifatun Nur, Sadia Afrin, Tashrif Mahmud Minhaz, Helena Khatoon

**Affiliations:** ^1^Department of Aquaculture, Faculty of Fisheries, Chattogram Veterinary and Animal Sciences University, Chattogram 4225, Bangladesh; ^2^Department of Genomics and Bioinformatics, Faculty of Biotechnology and Genetic Engineering, Chattogram Veterinary and Animal Sciences University, Chattogram 4330, Bangladesh

**Keywords:** antioxidant, bialgal, biochemical, immunity, survival, unfed

## Abstract

Diseases, overfishing, and habitat loss are constantly reducing oyster populations. Moreover, environmental pollution and natural disasters also hinder offshore shellfish cultivation. Therefore, filter-feeding bivalves could be cultivated on land-based system to maintain the culture condition and secure food safety. Four different diets including *Chaetoceros gracilis* (CG), *Tetraselmis chuii* (TC), mixture of CG and TC (CG/TC), and fresh seawater without feed (Con) were trialed for *Crassostrea belcheri* spat in this research. After 35 days of culture, the highest survival rate (SR), volume, and weight increment (WI) of oyster and improved water quality appeared in CG/TC. In the same manner, CG/TC diet exhibited greater lipase (LPS), pepsin (PES), catalase (CAT), alkaline phosphatase (AKP), and hydrogen peroxide (H_2_O_2_) activity. Conversely, amylase (AMS), acid phosphatase (ACP), superoxide dimutase (SOD), malondialdehyde (MDA), and lysozymes (LZMs) activities were substantially higher in CG diet compared to TC, CG/TC, and Con. No significant differences were observed among CG, TC, and CG/TC for antioxidant capacity (AOC). In this investigation, mixed algal diet had excellent results for the growth and development of oyster, whereas unialgal diet improved immunity and AOC to survive in unfavorable conditions. This observation help elucidates the knowledge on microalgal diet influenced immune modulation and health of marine bivalves in the scenario of land-based farming.

## 1. Introduction

There are more than 20,000 bivalve species found throughout the world, and most of them (includes oysters, scallops, clams, and so on) possess substantial economic and scientific significance [1]. Among bivalve's oysters are widely distributed and considered as one of the most valued seafood in the world. In Bangladesh, there is a domestic market demand of oyster meat and shell along with export opportunities [2]. Aquaculture plays a crucial role to meet the demand, consumption, and use of oyster today, making it highly significant for the local fishing economies [3].

Due to overfishing, habitat destruction, and diseases, the oyster populations have been declining day by day [4]. In addition, the major obstacles in traditional offshore bivalve culture are natural disasters and environmental pollution. Like fish and shrimp, the cultivation of bivalve species should shift to indoor culture in order to have better control over the culture condition and secure food safety. Nevertheless, the availability of food in this particular situation is minimal [5]. Microalgae serve as the primary food source for bivalves, providing a wide range of nutrients to facilitate their growth and development [6]. For bivalve larvae, suitable microalgal diet determined by some factors which include size, ease of culture, absence of toxicity, and the larvae's ability to capture, consume, digest, and absorb the algae [7]. Despite the established importance of microalgae, there is limited comprehensive research on how different microalgal diets influence the growth performance and physiological responses of oysters, particularly under controlled indoor aquaculture conditions.

Digestive enzymes play an important role in metabolic reactions, consequently their activity is frequently used as indicators of an organism's nutritional state [8]. The enzymes amylase (AMS), pepsin (PES), and lipase (LPS) have a potential impact on growth performances [9, 10]. Alkaline phosphatase (AKP), superoxide dismutase (SOD), and catalase (CAT) serve as nonspecific humoral immune components and play a vital role in immune responses [11]. The fundamental activities of antioxidant and immune systems involve the elimination of active oxygen (O_2_^−^) and foreign particles. This is performed through the actions of various enzymes and molecules, such as SOD, CAT, and lysozyme (LZM). The organism may experience oxidative stress when the production rate of reactive oxygen species is high, that can enhance antioxidant responses. Currently, the assessment of oxidative stress and damage in bivalve often involves measuring the antioxidant activities. This acts as a biomarker to indicate the overall health condition of these organisms [12, 13].

However, there is limited data on dietary microalgal effects on growth, digestive enzyme activity, and biochemical assays of oysters. Thus, this study aimed to determine the effects of microalgal diets on the growth, water quality, digestive enzyme, immunity, and antioxidant capacity (AOC) of edible oyster *Crassostrea belcheri*. The findings indicate the health and physiological condition of oysters when they fed to three different microalgal diets and one unfed diet. We hypothesize that dietary microalgae significantly modulate growth, oxidative stress, and physiological resilience, contributing to improved health and performance in oysters. This research provides novel insights into technical aspects, which include the sustainable cultivation of bivalves to contribute for the development of blue economy.

## 2. Materials and Methods

### 2.1. Algal Diet Culture

Pure microalgal isolates of *Chaetoceros gracilis* (CG) and *Tetraselmis chuii* (TC) were collected from the live feed research corner of Chattogram Veterinary and Animal Sciences University. The selection of CG and TC as the algal diet was based on their nutritional profiles, which are rich in essential fatty acids and proteins required for optimal growth and health of bivalve species [14, 15]. Those algal cells were cultured in sterilized seawater using conway medium [16]. For optimum growth of microalgae, light intensity (100 μEm^−2^s^−1^), continuous aeration rate (4.53 ± 0.53 mg/L), and temperature (24 ± 1°C) were maintained at the microalgae culture unit in the hatchery of Coastal Biodiversity, Marine Fisheries and Wildlife Research Center, Cox's Bazar. The algal cell densities were measured by counting cells using Neubauer hemocytometer under a light microscope (OPTIKA B 150, Italy, magnification of 40x) on regular basis specifically every 24 h during the culturing period to ensure consistent monitoring of algal growth.

### 2.2. Oyster Husbandry

The experimental oyster *C. belcheri* spats (average length in mm: 32.42 ± 0.93) were collected from the oyster culture site Nazirartek, Cox's Bazar at the middle of November, 2023. Then spats were immediately transferred to laboratory and placed into a rectangular tank (capacity = 80 L, size = 66 cm × 48 cm × 37.5 cm) filled with 26 ppt filtered and aerated seawater. Oysters were acclimated for 3 days to adapt in the laboratory conditions and no feed was given on this period. Continuous aeration (5.78 ± 0.52 mg/L) was provided inside each for the even distribution of microalgal diet. Daily, 50% of experimental water volumes were exchanged manually with freshly prepared seawater prior to each feeding.

### 2.3. Experimental Diets and Feeding

Four different diets were given to the oyster. Each dietary groups were set up in triplicate and placed in durable tank contained 60 L seawater (*n* = 30). The study period was 35 days (November~December, 2023). During the experiment, total three microalgal diet including 100% CG, 100% TC, mixture of 50% CG and TC were given in “CG,” “TC,” and “CG/TC” treatment, respectively. Fourth experimental group was only filled with fresh seawater without feed/baits as the control (Con). The temperature (21.35 ± 0.45°C) was consistently maintained across all treatment groups. The oysters were fed twice daily 12 h apart (07.00 AM and 07.00 PM) at a rate of 5 × 10^6^ microalgal cells/mL for 35 days. Feed intake was indirectly monitored by measuring the concentration of microalgal cells in the water before and after each feeding. The quantity of diet was determined through the Neubauer hemocytometer ensuring the desired concentration of microalgae was achieved.

### 2.4. Survival Rate (SR)

After ending the experiment, total number of live oysters of each replication was measured for determination of SR. Survivability was calculated by using the following equation [17]:

(1) $$\begin{array}{c} \frac{\text{The number of live individuals at the end of the experiment}}{\text{The number of live individuals at the beginning of the experiment}}\times 100\text{\% }. \end{array}$$

### 2.5. Growth Performance

An electronic balance (Radwag PS 1200.R2, Poland, 0.01 g precision) was used to quantify the total weight gain and digital slide calipers (0.02 mm accuracy) was used to measure the increments of length, width, and thickness in the beginning and ending of the experiment. Whole oyster volume and shell volume were determined by the method described by Damar et al. [18]. Oyster volume increment, shell volume increment, length increment (LI; mm), and weight increment (WI; g) were determined by subtracting the final value from initial.

### 2.6. Edible Percentage (EP)

The EP of oyster *C. belcheri* was ascertained by analyzing the ratio of the meat weight to the oyster whole weight [19]:

(2) $$\begin{array}{c} \text{EP}=\text{ Meat wet weight}/\text{oyster total weight}\times 100. \end{array}$$

### 2.7. Determination of Water Quality Parameter

Physical parameters including water temperature, pH, dissolved oxygen (DO), salinity, total dissolved solids (TDSs), and electrical conductivity (EC) of each tank were measured regularly in the experimental period by using a multiparameter digital probe (HANNA, HI98494). The probe was calibrated before each use following the manufacturer's guidelines. In addition, chemical parameters such as total ammonium nitrogen (TAN), nitrite-nitrogen (NO_2_─N), and soluble reactive phosphorus (SRP) were determined on laboratory by chemical method [20].

### 2.8. Determination of Digestive Enzyme Activities and Biochemical Assays

Digestive enzyme, immune-response, and antioxidant activities were evaluated by commercial test kits (Nanjing Jiancheng Bioengineering Institute, Jiangsu, China) as per the manufacturers' instructions [21, 22]. After 35 days of cultivation, eight digestive gland tissues were randomly sampled from each replicate for enzymatic and biochemical analysis. The tissues were homogenized, and the resulting homogenates were centrifuged at 10,000 × *g* (equivalent to 10,000 rpm) for 20 min at 4°C using a high-speed refrigerated centrifuge. The supernatant was then collected and transferred into 2.0 mL tubes, with all assays performed within 24 h of extraction.

The total protein (TP) concentration was measured using the Total Protein Quantitative Assay Kit (Coomassie Brilliant Blue Method; Catalog No. A045-2). PES activity was assessed with the Pepsin Assay Kit (Catalog No. A080-1-1), while LPS activity was analyzed using the Lipase Assay Kit (colorimetric method; Catalog No. A054–1-1). Likewise, AMS activity was evaluated with the Amylase Assay Kit (Starch–iodine colorimetric method; Catalog No. C016–1-1).

For biochemical assays, CAT activity was analyzed using the Catalase Assay Kit (colorimetric method; Catalog No. A007–1-1), while SOD activity was assessed using the Superoxide Dismutase Assay Kit (WST-1 method; Catalog No. A001–3-2). The malondialdehyde (MDA) content was determined using the Malondialdehyde Assay Kit (TBA method; Catalog No. A003–1-2), and hydrogen peroxide (H_2_O_2_) levels were measured with the Hydrogen Peroxide Assay Kit (spectrophotometric method; Catalog No. A064-1-1).

Enzymatic assays for AKP and acid phosphatase (ACP) were conducted using the AKP Assay Kit (colorimetric method; Catalog No. A059-1-1) and ACP Assay Kit (colorimetric method; Catalog No. A060-1-1), respectively. The AOC was measured with the Antioxidant Capacity Assay Kit (colorimetric method; Catalog No. A015-1). Finally, LZM activity was analyzed using the Lysozyme Assay Kit (turbidimetric method; Catalog No. A050-1-1). All assay kits used for the measurements in this study were sourced from Jiancheng Bioengineering Institute, Nanjing, China.

### 2.9. Statistical Analysis

Assumptions of normality and homogeneity of variances were tested for all data by using Shapiro–Wilk test and Levene's test statistics. The results of the SR, growth, water quality, enzymatic activities data, and biochemical assays were expressed as mean ± standard deviation (SD), and the significant differences (*p* < 0.05) among diets were determined using one-way ANOVA and Tukey's HSD post hoc tests. All analyses were conducted using R version 4.1.3. A correlation analysis was carried out between various parameters related to water quality and growth parameters.

## 3. Results

### 3.1. Growth Parameter

Growth parameters among four different treatments are depicted in Table 1. The highest and lowest SR was found in CG/TC treatment (96.7% ± 3.34%) and Con treatment (85.6% ± 1.92%), respectively, rather than other treatments. Compared to other treatments, this study demonstrated a significant (*p* < 0.05) increase in oyster volume increment, shell volume increment, LI, weight gain rate, and EP following the CG/TC treatment.

### 3.2. Physical Parameter

Physical parameters, includes temperature, DO, pH, salinity, and EC data of four experimental diet groups are represnted in Table 2. All of the parameters were within the standard range for oyster culture [1, 23]. The physical parameters did not show significant (*p* > 0.05) differences among the treatments during the experiment.

### 3.3. Chemical Parameters

**Figure 1:**
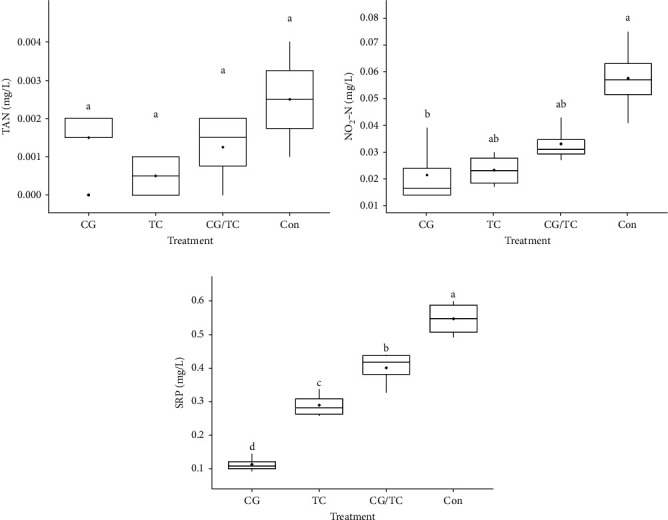
(A) Total ammonium nitrogen (TAN), (B) nitrite-nitrogen (NO_2_-N), and (C) soluble reactive phosphorus (SRP) concentrations in different treatments during the experimental period. Vertical error bars show the standard deviations (SDs; *n* = 3). Same superscript letters are not significantly different (*p* > 0.05). Here, “CG” = *Chaetoceros gracilis, “TC” = Tetraselmis chuii, “CG*/TC” = mixture of CG and TC, and “Con” = fresh seawater without feed/baits.

Chemical parameters including SRP and NO_2_─N displayed significant (*p* < 0.05) differences among all treatments (Figure 1). SRP and NO_2_ concentrations were lower in the CG treatments than in the other treatments, whereas SRP and NO_2_ values were 0.11 ± 0.02 and 0.024 ± 0.01 mg/L, respectively. The control group reported the highest values, with SRP 0.547 ± 0.05 mg/L and NO_2_─N 0.048 ± 0.01 mg/L.

### 3.4. Environmental Factors Quality and Growth Performance of Edible Oyster

**Figure 2:**
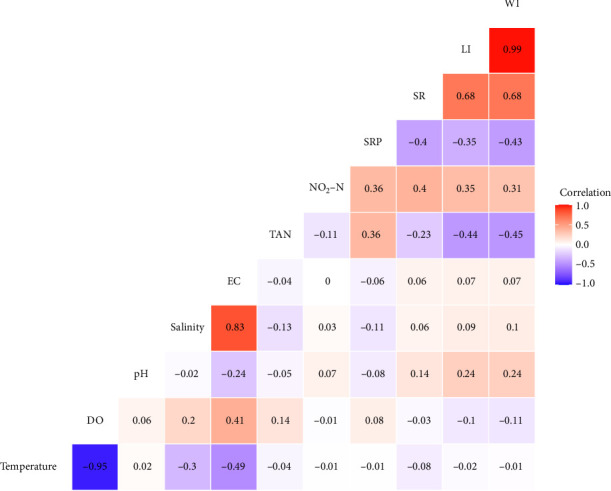
Relationship among the water quality and growth parameters of *Crassostrea belcheri* in indoor culture system. Here, the full forms of the parameters are: temperature (°C); salinity (psu); dissolved oxygen (DO, mg/L); electrical conductivity (EC; µScm^−1^); total ammonia nitrogen (TAN; mg/L); nitrite nitrogen (NO_2_─N; mg/L); soluble reactive phosphorus (SRP; mg/L); length increment (LI; mm); weight increment (WI; g); survival rate (SR; %). The values given on each axis represent the range of each individual parameter. Correlation coefficients (*r*) are represented by numerical values, with the font size of *r* increasing as the correlation becomes stronger. The significance levels (*p*) are indicated by dark color (*n* = 12).

The findings revealed that pH and NO_2_─N were positively correlated and TAN and SRP were negatively correlated with LI and WI of edible oyster. Other environmental parameters such as temperature, salinity, and DO did not find to be correlated with the LI and WI of oyster. For SR, TAN and SRP showed negative correlation, whereas NO_2_─N exhibited positive correlation (Figure 2).

### 3.5. Digestive Enzyme Activities

**Figure 3:**
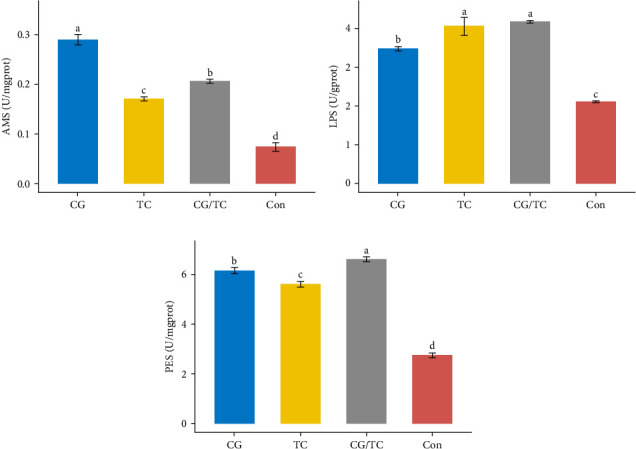
Activities of digestive enzyme of *Crassostrea belcheri* fed on different diets. (A) Amylase (AMS), (B) lipase (LPS), and (C) pepsin (PES). Bars are expressed as means ± standard deviation (SD; *n* = 3). Significant differences among the CG, TC, CG/TC, and Con groups are indicated by distinct letters (one-way ANOVA, Tukey's test, *p*  < 0.05). Here, “CG” = *Chaetoceros gracilis*, “TC” = *Tetraselmis chuii*, “CG/TC” = mixture of CG and TC, and “Con” = fresh seawater without feed/baits.

The digestive enzyme activity of four treatments in the digestive gland of edible oysters is displayed in Figure 3. The CG/TC treatment achieved significantly (*p* < 0.05) higher LPS and PES activity than other treatment groups. However, when contrasted to other treatment groups, a significant (*p* < 0.05) rise indicated the highest level of AMS activity in terms of CG treatment, which was 0.290 ± 0.01 U/mgprot.

### 3.6. Biochemical Assays

**Figure 4:**
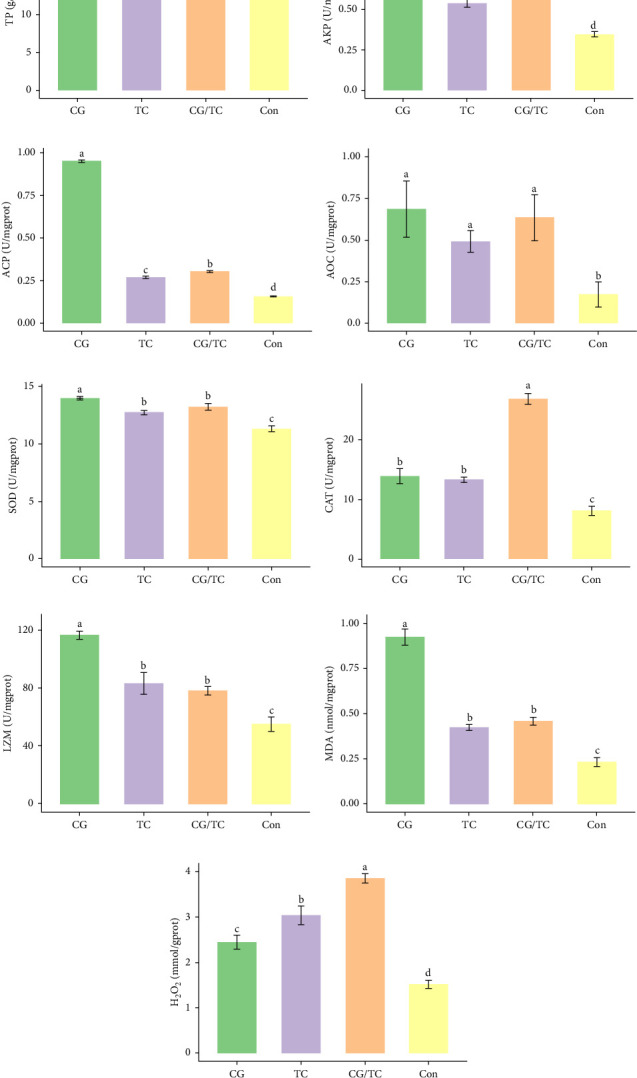
Activities of immune and antioxidant enzymes of edible oyster, *Crassostrea belcheri* in different treatments. (A) Total protein (TP), (B) alkaline phosphatase (AKP), (C) acid phosphatase (ACP), (D) antioxidant capacity (AOC), (E) superoxide dimutase (SOD), (F) catalase (CAT), (G) lysozymes (LZMs), (H) malondialdehyde (MDA), and (I) hydrogen peroxide (H_2_O_2_). Bars are expressed as means ± standard deviation (SD; *n* = 3). Significant differences among the CG, TC, CG/TC, and Con groups are indicated by distinct letters (one-way ANOVA, Tukey's test, *p*  < 0.05). Here, “CG” = *Chaetoceros gracilis*, “TC” = *Tetraselmis chuii*, “CG/TC” = mixture of CG and TC, and “Con” = fresh seawater without feed/baits.

Edible oyster fed four different types of diets related immunological and antioxidant enzymes TP, AKP, ACP, AOC, SOD, CAT, LZM, MDA, and H_2_O_2_ are illustrated in Figure 4. In terms of CAT, AKP, and H_2_O_2_ activity, the CG/TC treatment reported significantly (*p* < 0.05) highest values than other groups. In addition, the values of ACP, SOD, MDA, and LZM fed on the CG diet treatment were significantly (*p* < 0.05) greatest. No significant (*p* > 0.05) differences was found among algae treatments, but differ with control group in case of AKP. Moreover, the TP activity was observed to be significantly (*p* < 0.05) higher in TP treatment (20.9 ± 0.77 g/L) and lower in control (17.9 ± 0.83 g/L), respectively.

## 4. Discussion

Edible oyster, *C. belcheri* is the most potential aquaculture species for the development of blue economy in near future due to its ecological role, high production, and simple culture method. The present study investigated the dietary effects of microalgae on the survivability, growth, water quality, digestive enzymatic activity, and biochemical assay of *C. belcheri* spat.

In this study, survival and growth rate reported significant (*p* < 0.05) differences among the four diets. Mixture of CG and TC (CG/TC) diet showed highest survivability and growth performance in contrast with CG, TC, and fresh seawater without feed/baits (Con) diet. Likewise, Krampah et al. [24] revealed that survival and growth performance showed better performance of *Crassostrea tulipa* larvae fed on mixed algal diet. Similar findings reported that mixed algal diet expressed better performances than monospecific diet in case of growth and survival in oyster larvae. Combined algal diet provide improved nutrition than single species diet [25, 26]. Furthermore, *Tisochrysis lutea* and *Chaetoceros calcitrans* combined diet resulted best growth rate for *Crassostrea gigas* [27].

Utilization of microalgae can be a cost-effective method for improving water quality in aquaculture [28]. The incorporation of a single species algal diet (CG or TC) and a combined diet (CG/TC) into the feed results in the enhancement and preservation of optimum water conditions throughout the duration of the culture. In this present research, there was no significant (*p* > 0.05) differences among physical parameters. Conversely, the water samples of algal diets exhibited a significant (*p* < 0.05) decrease in NO_2_─N and PO_4_─P levels. This finding aligns with the outcomes of Guedes and Malcata [29], which shown that microalgae have the ability to stabilize and enhance the water quality of a culture. In the same way, Chen et al. [30] reported that TC exhibited the greatest uptake of TAN, which results in reduced nutrient levels towards the end of the culture period in comparison to the other species of microalgae examined. These results are consistent with the findings reported by Rahman et al. [31]. Those outcomes were line up with the present research findings.

The enzymatic activity in the digestive gland plays an important role in the digestion and absorption of feed. This process is strongly influenced by the feed quality and composition [32]. Digestive enzyme activity is used as an indicator of digestion processes and nutritional condition of an organism [9]. In such manner, AMS and LPS activity elevated the growth performance of organisms [10]. In this investigation, AMS and PES activity were highest in CG and CG/TC treatment, while LPS activity was greater in both TC and CG/TC treatment. According to Zhu et al. [33], *Chaetoceros muelleri* diet showed better AMS and PES activity in manila clam, *Ruditapes philippinarum*. The outcomes also demonstrated that razor clam *Sinonovacula constricta* has improved AMS and LPS activity with *C. muelleri* diet. Result of the study showed that, in case of unfed diet, the activity of AMS, LPS, and PES was lowest in this study. This results align with the findings of Zhu et al. [33].

Shellfish species lack an acquired immune system so their defense mechanisms primarily depend on nonspecific immune responses [34]. The AOC of an organism involves both enzymatic and nonenzymatic antioxidant activities. Enzymes such as AOC, CAT, SOD, and MDA play a role in protecting against free radicals. The AOC level is an indicator of the ability to resist oxidation and is linked to the health condition of aquatic animals [35]. Furthermore, ACP serves as a bioindicator for lysosomes and enhances the process of phagocytosis and degradation of foreign substances in an acidic environment. LZM is abundant in the bloodstream and blood cells of various animals and has a significant impact on immune function [36, 37]. The activity of SOD, which constitutes the first line defense mechanism against reactive oxygen species, is intricately linked to the immune response of organisms [38]. The present study revealed that CAT activity of oyster was maximum in CG/TC treatment and minimal in Con. That means combined microalgal diet positively influenced the immune activity. The findings line with the study of Amaro et al. [39], where dietary mixture of 75% microalgae and 25% macroalgae showed better CAT in pacific oysters. Similarly, the study of Sun et al. [40], disclosed that the activities of AKP, SOD, and CAT are prominent in triangle sail mussels *Hyriopsis cumingii* fed with *Cyclotella* sp. treatment.

In this research, the edible oysters in CG appeared higher ACP, SOD, LZM, and MDA content and considerably lower AKP and H_2_O_2_ content compared with other groups. In case of AOC, all microalgal diet showed similar result where no significant differences were found (*p* > 0.05). The diet CG can boost stress tolerance of edible oyster for the adaptation in the unfavorable conditions or pathogens. Single species diatom enriched diet exhibited better immunity and AOC. Studies suggest that diatoms can enhance immunity by promoting immune tolerance to various microbial and viral pathogens, thereby, creating an effective defense against diseases in aquatic animals [41]. In the same way, *Chaetoceros calcitrans* exhibited beneficial effect on the structure of the intestines, the immune response, the proportion of granulocytes, and the weight balance of the bivalve species *C. gigas* [42].

The improved AOC in the CG/TC group can be attributed to the complementary nutritional profiles of CG and TC. CG provides essential fatty acids like EPA, which enhance antioxidant responses, while TC is rich in carotenoids and bioactive compounds that combat oxidative stress [43, 44]. Their combination likely created a synergistic effect, optimizing nutrient intake, and boosting key antioxidant enzymes like CAT and AOC. This synergy aligns with previous studies showing that mixed algal diets enhance immune and antioxidant activity in bivalves by providing diverse and balanced nutrition [14, 45, 46].

## 5. Conclusion

This study is the first to analyze metabolism, immunity, and AOC of edible oyster *C. belcheri* fed with different microalgal diet. Edible oyster fed with combined algal diet proved better survival and growth. However, immunity, AOC, and metabolism performance of oyster fed unialgal diatom suppressed mixed algal diet. The outcomes of the study provide basic information regarding the growth, water quality, digestion, and biochemical assay through which algal diets enhance the overall health of bivalve under controlled conditions. However, this research investigated the immune and antioxidant responses of digestive glands in oysters over a short period of time. Therefore, further research is needed to analyze the long-term effects on feeding behavior, survival, and reproductive performance. This will provide more insights into the ecotoxicological aspects of the health and bioremediation of this species.

## Figures and Tables

**Table 1:** Different growth parameters of *Crassostrea belcheri* fed with experimental diets.

Parameter	Treatment			
	CG	TC	CG/TC	Con
Oyster volume increment (cm^3^)	0.41 ± 0.01^b^	0.44 ± 0.13^a,b^	0.46 ± 0.00^a^	0.33 ± 0.02^c^
Shell volume increment (cm^3^)	0.26 ± 0.01^b^	0.28 ± 0.02^a,b^	0.30 ± 0.01^a^	0.220 ± 0.02^c^
LI (mm)	1.13 ± 0.13^b^	1.48 ± 0.09^a^	1.71 ± 0.05^a^	0.56 ± 0.08^c^
WI (g)	0.38 ± 0.03^b^	0.46 ± 0.04^a,b^	0.52 ± 0.04^a^	0.20 ± 0.02^c^
Weight gain rate (%)	5.76 ± 0.54^b^	7.22 ± 0.43^a^	7.87 ± 0.48^a^	2.83 ± 0.14^c^
EP (%)	7.10 ± 0.17^c^	9.28 ± 0.30^b^	10.3 ± 0.32^a^	5.09 ± 0.13^d^

*Note:* Values are represented as means ± SD (*n* = 3). Within the row, means with same superscript letters are not significantly different (*p*  > 0.05). Con, fresh seawater without feed/baits.

Abbreviations: CG, *Chaetoceros gracilis*; CG/TC, mixture of CG and TC; EP, edible percentage; LI, length increment; SD, standard deviation; TC, *Tetraselmis chuii*; WI, weight increment.

**Table 2:** Physical parameters of different treatments during the experiment.

Treatment	Physical parameters				
	Temperature (°C)	DO (mg/L)	pH	Salinity (psu)	EC (µScm^−1^)
CG	21.5 ± 2.60	5.51 ± 0.34	8.21 ± 0.06	25.9 ± 1.43	39.6 ± 2.95
TC	21.4 ± 2.65	5.50 ± 0.33	8.22 ± 0.06	25.9 ± 1.54	39.6 ± 3.13
CG/TC	21.4 ± 2.66	5.51 ± 0.33	8.24 ± 0.05	26.0 ± 1.51	39.7 ± 3.02
Con	21.6 ± 2.55	5.60 ± 0.41	8.19 ± 0.12	25.6 ± 1.80	39.1 ± 3.49

*Note:* Values are represented as means ± SD (*n* = 3) and are not significantly different (*p*  > 0.05). Con, fresh seawater without feed/baits.

Abbreviaions: CG, *Chaetoceros gracilis*; CG/TC, mixture of CG and TC; DO, dissolved oxygen; SD, standard deviation; TC, *Tetraselmis chuii*.

## Data Availability

The data that support the findings of this study are available from the corresponding author upon reasonable request.
